# Subpubic cartilaginous cystic lesion presenting as a vulvar mass: a case report

**DOI:** 10.1186/1752-1947-3-7294

**Published:** 2009-05-29

**Authors:** RJW Hoogendoorn, HWM Kayser, JJ Weening, AAW van Geloven

**Affiliations:** 1 Department of Orthopaedic Surgery, VU University medical center, Amsterdam, The Netherlands; 2 Department of Radiology, Tergooiziekenhuizen, Hilversum, The Netherlands; 3 Department of Pathology Tergooiziekenhuizen, Hilversum, The Netherlands; 4 Department of Surgery, Tergooiziekenhuizen, Hilversum, The Netherlands

## Abstract

**Introduction:**

A subpubic cartilaginous cyst is a rare and innocent defect originating from the symphysis pubis and may present as a vulvar mass and/or chronic abdominal pain. The symphysis pubis is a non-synovial amphiarthrodial joint that forms a fibrocartilaginous union between the two pubic bones and in general lies without mainstream interest, despite its clinical relevance. This case report focuses attention on this joint.

**Case presentation:**

A 55-year-old Caucasian woman presented with a painful and rapidly increasing vulvar mass. Imaging techniques revealed a cyst-like structure originating from a degenerated symphysis pubis. The cyst was excised, however, recovery was complicated by a symphysiolysis.

**Conclusion:**

A subpubic cartilaginous cyst is a rare cystic lesion originating from the symphysis of the pubic bone which presents as a vulvar mass which might be complicated by symphysiolysis. Several diseases affect the symphysis pubis and result in significant discomfort and disability in patients. The symphysis pubis is therefore clinically important and should not be omitted in the differential diagnosis of a vulvar mass and/or chronic abdominal pain.

## Introduction

The subpubic cartilaginous cyst (SCC) is a rare cystic lesion originating from the symphysis of the pubic bone which presents as a vulvar mass and/or chronic abdominal pain [1]. These lesions consist of a collagenous capsule containing gelatinous and degenerating fibrocartilaginous tissue, acellular debris and mucin [2]. Furthermore, these lesions are associated with the pubic bone [2]. Diagnosis of this lesion is based on radiological examination i.e. ultrasonography, or computed tomography (CT) or magnetic resonance imaging (MRI) and histologic biopsy [3]. The symphysis pubis is a non-synovial amphiarthrodial joint that forms a fibrocartilaginous union between the two pubic bones. Because it seldom produces dramatic symptoms or signs, this articulation often falls outside the mainstream interest. Yet this joint is not without clinical significance [4]. Based on our patient with SCC, we would like to draw attention to the symphysis pubis and its clinical relevance.

## Case presentation

A 55-year-old woman, G4P3, presented to her physician with a painful vulvar mass that had developed over approximately six months. The mass had suddenly became painful after rising, resulting in an antalgic walking pattern for three days. After this, the pain had subsided spontaneously, however, the mass increased more rapidly. The patient was not on any medication. Her medical history revealed carpal tunnel syndrome, ganglion cyst in the foot, and vaginal uterus extirpation. Physical examination revealed a solitary vulvar mass at the cranial site of the vulva, directly under the clitoris. The mass was round, smooth and was connected to the symphysis pubis. There was no connection to the mucous membrane of the vulva. There were no palpable masses in the groins.

**Figure 1:**
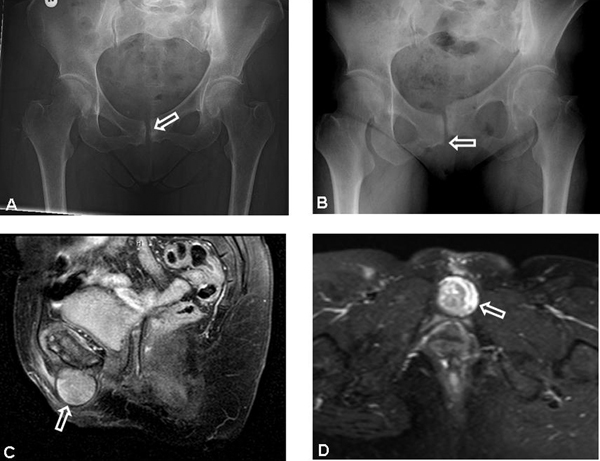
**(A) Original pelvic X-ray obtained before surgery**. The symphysis pubis is wide and has a sclerotic aspect indicating the degenerative aspect of the joint. **(B)** Postoperative pelvic X-ray (one leg) clearly indicating the symphysiolysis that developed postoperatively. **(C)** Sagittal T2 weighted magnetic resonance image of the subpubic degenerative cyst, showing the size of the cyst (3.6 × 3.7 × 3.5 cm). **(D)** Transversal T2 weighted magnetic resonance image of the subpubic degenerative cyst demonstrating the characteristic heterogenic hyperintense aspect of the cyst and the origin from the symphysis pubis.

Subsequently, ultrasonography was performed which revealed a 3.7 × 2.9 × 4.0 cm large mass which was in a close relationship with the symphysis pubis. Conventional X-ray examination of the pelvis showed a relatively wide symphysis pubis with a sclerotic aspect (Figure 1A). A cortical defect was also noted on the cranial side of the inferior ramus of the pubis. Other abnormalities were not observed. In addition, MRI of the pelvis was performed using intravenous gadolinium contrast. The mass measured 3.7 × 3.6 × 3.5 cm and was well defined from the surrounding structures. The mass had a low signal intensity on the T1-weighted sequences and a heterogeneous aspect on the T2-weighted images (Figure 1C and 1D). Gadolinium uptake was not observed in the mass itself, however, the border demonstrated some marginal uptake. Also, some edematous changes were observed in both the left as well as the right inferior ramus of the pubis. Based on these findings, the mass was identified as benign, more accurately, a ganglion cyst of the symphysis pubis.

**Figure 2:**
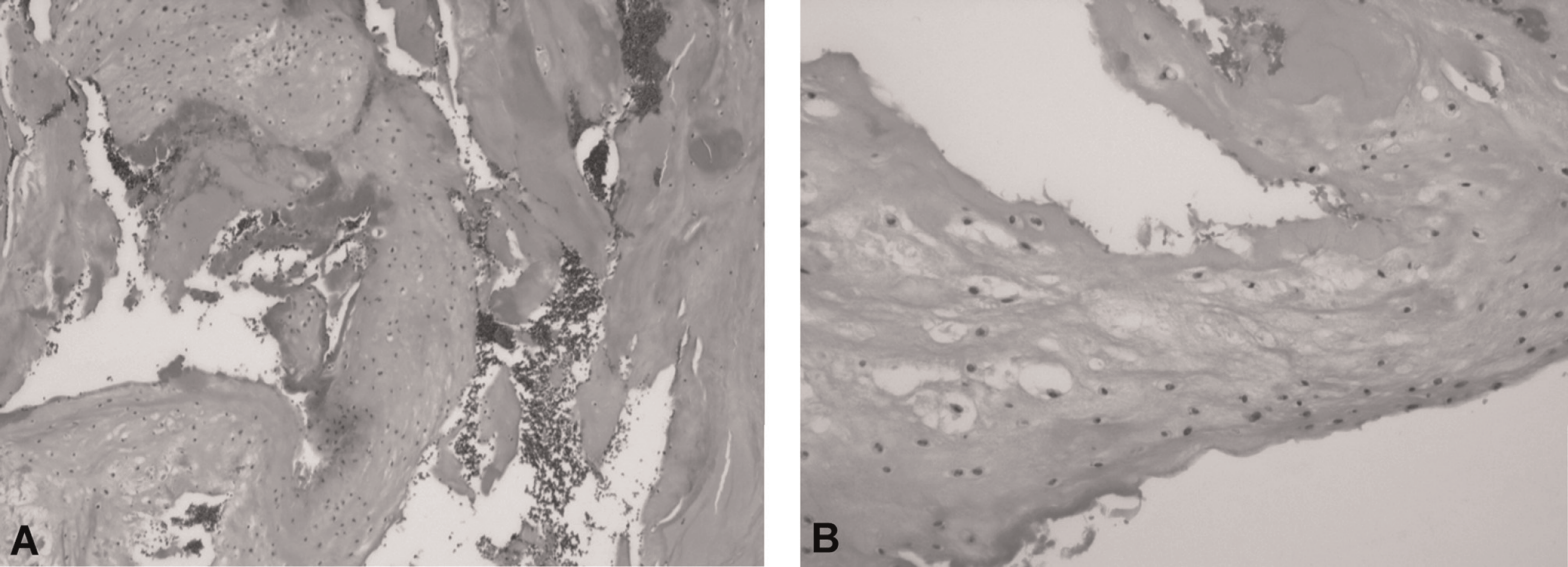
**(A) Microscopic section (hematoxylin and eosin staining, 100×) of the content of the degenerative cyst**. In this section, the degenerative fibrocartilaginous content of the cysts can be observed, including focal disintegration. **(B)** Microscopic section (hematoxylin and eosin staining 200×) of the content of the degenerative cyst. In this section, the fibrocartilaginous tissue is depicted in more detail.

Aspiration and deposition of local steroids was attempted once with a 16 G needle: this resulted in a temporary reduction of the mass, however, the mass did not disappear. Subsequent aspiration was no longer possible, as no fluids could be withdrawn. Next, resection of the cyst was performed under general anesthesia, revealing a cyst containing yellow, granular debris and some serous fluids (Figure 2A and 2B). The mass originated from the symphysis and was resected from within the symphysis. Initially, the patient recovered well from the surgery and recurrence has not occurred during the follow-up. However, two months after surgery, the patient returned with a clicking, painful sensation in the pubic area, which was worst in the morning and directly after standing up. An X-ray revealed a symphysiolysis (Figure 1B). This was treated conservatively.

Histopathologic analysis of the resection specimen revealed degenerated cartilage and synovial and connective tissue, surrounded by a connective tissue structure identified as the wall of the cyst (Figure 2A and 2B).

## Discussion

A subpubic cartilaginous cyst is a rare and poorly understood form of a ganglion cyst, arising from the symphysis pubis which was first described in 1996 [1]. Four cases have been reported before in the literature, where multiparous women presented either with a vulvar mass [1,2] or with chronic abdominal pain [3]. These cysts are described as well-circumscribed cystic masses containing fibrocartilaginous tissue with extensive disintegration and mucinous cystic degeneration [1]. SCC is thought to originate from the pubic symphysis and to be degenerative in nature, as these cysts are encountered in multiparous women and are generally accompanied by narrowing, subchondral sclerosis and marginal spur formation of the symphysis pubis [3]. Histologically, the fibrocartilage of the pubic symphysis undergoes similar mucinous degenerative changes as observed in the SCCs, particularly in multiparous women [1]. In these women, clefts and cavities are observed in the pubic symphysis, which might cause the cyst formation.

Diagnosis is based upon clinical examination and radiological evaluation. The radiological appearance of the SCC depends on the mucinous material and chondrocytic elements in the contents. This results in a dense presentation on ultrasonography, a high density in CT images and a heterogeneous intensity on T1 and T2 MRI sequences [3].

The treatment of choice is resection, as aspiration is not possible due to the bulky content of the cysts and recurrences have not been reported in the literature although follow-up was limited (three years) [1]. In one case, the SCC was not treated and follow-up revealed no changes in size or characteristics after two years [3].

The resection of the SCC was complicated by a symphysiolysis (see Figure 1B). In general, symphysiolysis is only described following high-energy trauma or after labor [5]. Furthermore, several cases of symphysiolysis have been reported in patients with rheumatoid arthritis [5]-[7]. The latter is thought to develop following chronic corticosteroid therapy combined with minor trauma and/or arthritic changes [5]-[7]. One case of spontaneous symphysiolysis has been reported where an underlying cause was not found [8]. In our patient, degenerative changes were visible in the symphysis pubis, as described before in other subpubic cartilaginous cysts [3]. Although these changes resemble the changes that occur in osteoarthritis and rheumatoid arthritis, they are also described as residual changes following delivery [9]. One case of non-traumatic symphysiolysis following multiple pregnancies has been reported [10]. The cartilaginous cyst originated from the central portion of the symphysis pubis, and protruded through the anterior part of the symphysis pubis, compromising its stability. Resection of the cyst probably further destabilized the joint resulting in the observed symphysiolysis, although the posterior part of the symphysis joint was left untouched. Conservative therapy for this complication is based on literature dealing with symphysiolysis in pregnancy, which is treated conservatively with reduced physical activity, a pelvic girdle or a corset. When this therapy fails, and in traumatic disruption, metallic internal fixation is indicated. The latter was not necessary in our patient, as conservative treatment resulted in the disappearance of the clicking sensation and pain relief. In our opinion, the pre-operative pain and cosmetic issues for the patient justify the surgical procedure and its concomitant risks such as instability.

The differential diagnosis considered in patients presenting with a vulvar mass includes lipomas, Nabothian's, Bartholin's, Gartner's or paratubal cysts [11]. Also, malignant masses, such as squamous cell carcinoma, Bartholin's gland carcinoma, basal cell carcinoma and vulvar melanoma, must be considered in the differential diagnosis [12]. In general, easy differentiation is possible due to differences in localization and radiological characteristics [3]. Other cysts originating from the symphysis pubis have been described before: Morita et al. described a pseudosynovial cyst from the symphysis pubis based on rheumatoid arthritis [13] and Verrall et al. reported on subchondral cyst formation in football players [14].

An SCC can present with pain without the clear presence of a vulvar mass [3]. This pain might be located in the lower back, the abdomen or the groin. The differential diagnosis of these complaints is extremely varied and includes disorders of the abdomen, the reproductive organs, urologic disorders and musculoskeletal disorders. However, this differential diagnosis lies beyond the scope of this article.

In general, the symphysis pubis is not often considered in the differential diagnosis of the abovementioned complaints. Besides the SCC, several other relevant pathologic processes of the symphysis may occur resulting in significant symptoms. These disorders can be subdivided into infectious, congenital, metabolic, inflammatory, traumatic or degenerative causes, for example, the SCC [4] and are summarized in Table 1.

**Table 1:** Pathology involving the symphysis pubis

Congenital	Infectious	Metabolic	Tumor
Exstrophy of the bladder	Arthritis	Renal osteodystrophy	Metastatic carcinoma
Cleidocranial dysostosis	Osteomyelitis	Hyperparathyroidism	Myelosclerosis
Dyggve-Melchior-Clausen syndrome	Osteitis pubis	Chondrocalcinosis	Multiple myeloma
	Ankylosing spondylitis	Hemochromatosis	Hodgkin's disease
	Reiter's syndrome	Ochronosis	Unicameral bone cyst
	Tuberculosis		Desmoplastic fibroma
			Hereditary multiple exostosis

*Gamble* et al. *The symphysis pubis. Anatomic and pathologic considerations. CORR 1986.*^4^

The most frequently occurring disorder of the symphysis pubis is symphysis pubis dysfunction (SPD). Multiple terms have been used for this unambiguously defined condition that is rather defined as pain in the symphysis pubis ranging from mild discomfort to severely debilitating pain [15]. SPD is thought to occur due to the physiological pelvic ligament relaxation and increased joint mobility that is seen in pregnancy [15] and the incidence has been reported to be as high as 16% in pregnant women [16]. Treatment is generally conservative and delivery is curative for the majority within six months.

The most common inflammatory disease of the symphysis pubis is osteitis pubis, a self-limiting, non-infectious inflammation of the symphysis following trauma [17], pelvic (urogenital) surgery [18] and childbirth [19]. It is thought to be an overuse syndrome considering its relationship to mechanical stress and is treated with rest, anti-inflammatory drugs and physical therapy [4]. The overuse might also result in degenerative changes and eventually SCCs, as described in our patient and in the literature regarding multiparity [1,2]. This inflammation can be difficult to distinguish from infectious causes of symphysis pubis arthritis or osteomyelitis, as both conditions are related to an increased sedimentation rate and changes in the roentgenograms [4]. In general, the latter is seen within three groups: children, the elderly who have undergone genitourinary manipulation and intravenous drug users. In the latter, the causative organism most commonly is *Pseudomonas aeruginosa*[20]. Treatment of infectious arthritis or osteomyelitis is more difficult compared to osteitis pubis and consists of biopsy and curettage combined with intravenous antibiotics. Skeletal tuberculosis is a rare cause of pyogenic infection of the symphysis pubis, but has been increasing recently in the Netherlands with immigration. It is treated similar to arthritis/osteomyelitis. Finally, radiographic changes have also been observed in patients with seronegative arthropathies. In severe cases, the joint undergoes complete bony ankylosis [21]. Furthermore, multiple rare causes of pain in the symphysis pubis have been described in the literature including congenital anomalies, tumors and several metabolic disorders (Table 1).

## Conclusions

We describe a patient with degenerative changes in the symphysis pubis resulting in a rarely described subpubic cartilaginous cyst and symphysiolysis. The diseases of the symphysis pubis are summarized in this article and their clinical relevance is underscored.

## Abbreviations

CT: computed tomography; MRI: magnetic resonance imaging; SCC: subpubic cartilaginous cyst; SPD: symphysis pubis dysfunction.

## Consent

Written informed consent was obtained from the patient for publication of this case report and any accompanying images. A copy of the written consent is available for review by the Editor-in-Chief of this journal.

## Competing interests

The authors declare that they have no competing interests.

## Authors' contributions

RH and NG performed the surgery and were major contributors to the manuscript. HK performed the radiological studies and reviewed the manuscript. JW performed the histological studies and reviewed the manuscript. All authors read and approved the final manuscript.
